# Peroral cholangioscopy-guided biopsy with novel biopsy forceps in comprehensive cancer genomic profiling for cystic duct carcinoma

**DOI:** 10.1055/a-2313-9930

**Published:** 2024-05-17

**Authors:** Yujiro Kawakami, Yoshiharu Masaki, Keisuke Ishigami, Takehiro Hirano, Ayako Murota, Shintaro Sugita, Hiroshi Nakase

**Affiliations:** 1 Department of Gastroenterology and Hepatology, Sapporo Medical University School of Medicine, Sapporo, Japan; 2 Department of Surgical Pathology, Sapporo Medical University School of Medicine, Sapporo, Japan

**Keywords:** Pancreatobiliary (ERCP/PTCD), ERC topics, Cholangioscopy, Endoscopic ultrasonography, Biliary tract, Tissue diagnosis

Recent studies have demonstrated the benefits of comprehensive cancer genomic profiling (CGP) for detecting potential targets for genotype-matched therapy in patients with biliary tract cancer 1 2 . While peroral cholangioscopy (POCS) enables tissue acquisition for diagnosis 3 , its utility for CGP of biopsy samples remains unclear. Herein, we report a case of cystic duct carcinoma where novel biopsy forceps under POCS proved useful for CGP.

A 70-year-old man presented to our hospital with upper abdominal pain. Contrast-enhanced computed tomography revealed a cystic duct tumor infiltrating the portal vein, common hepatic artery, and celiac artery ( Fig. 1 ). Endoscopic retrograde cholangiopancreatography demonstrated obstruction of the cystic duct ( Fig. 2 ). Owing to difficult insertion for fluoroscopy-guided biopsy, POCS was performed using the Spy-Glass Direct Visualization System (SpyGlass DS; Boston Scientific, Marlborough, Massachusetts, USA), revealing a cystic duct mass with irregularly dilated and tortuous blood vessels ( Fig. 3 **a** ). POCS-guided targeted biopsies ( Fig. 3 **b,c** ) were subsequently performed using the SpyBite MAX forceps (SpyBite MAX; Boston Scientific) ( Fig. 4 , Video 1 ).

**Fig. 1:**
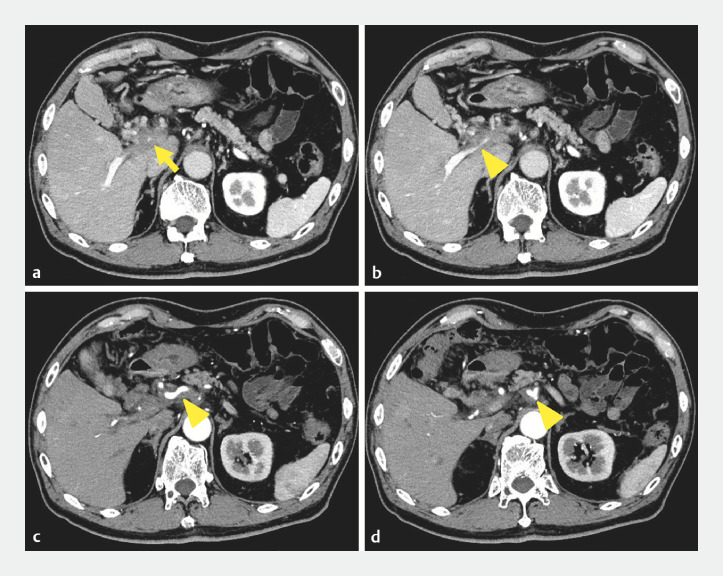
Contrast-enhanced computed tomography images ( **a–d** ), revealing cystic duct tumor ( **a** ) (arrow), infiltrating the portal vein ( **b** ), common hepatic artery ( **c** ), and celiac artery ( **d** ) (arrowheads).

**Fig. 2:**
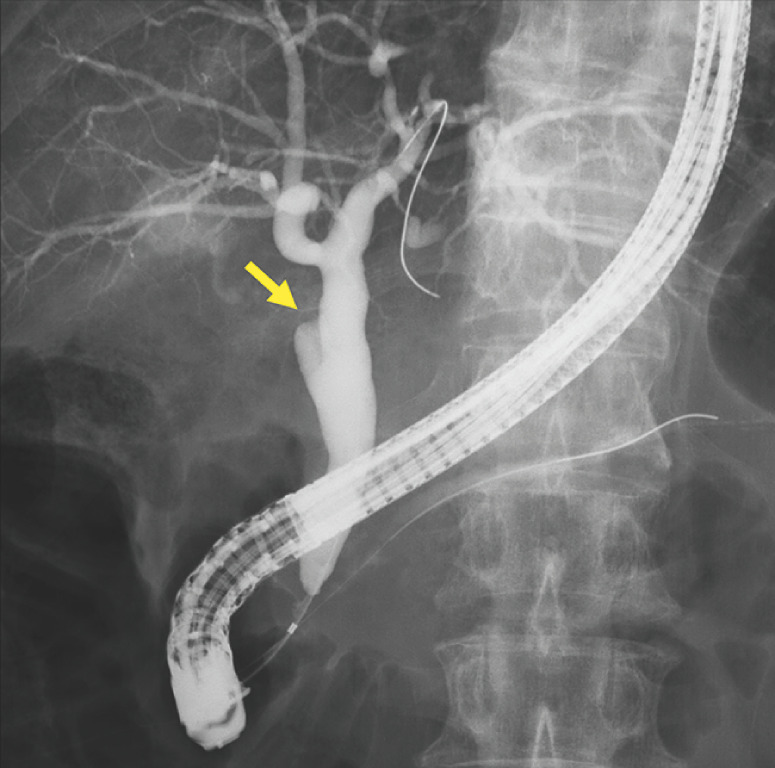
Endoscopic retrograde cholangiopancreatography demonstrated a cystic duct stricture (arrow).

**Fig. 3:**
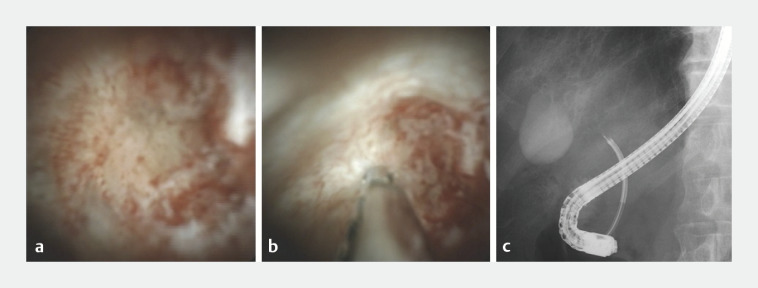
Cholangioscopy and fluoroscopy. **a** Peroral cholangioscopy (POCS) revealed a cystic duct mass with irregularly dilated and tortuous blood vessels. **b, c** A POCS-guided biopsy was performed using the SpyBite MAX biopsy forceps (Boston Scientific, Marlborough, Massachusetts, USA).

**Fig. 4:**
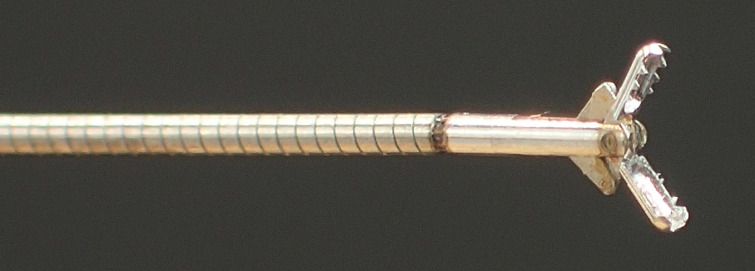
The SpyBite MAX (Boston Scientific, Marlborough, Massachusetts, USA).

Peroral cholangioscopy-guided biopsy using novel biopsy forceps for the comprehensive cancer genomic profiling of cystic duct carcinoma.Video 1

Histopathology revealed adenocarcinoma ( Fig. 5 ). Based on the radiological and pathological findings, we diagnosed the patient with unresectable cystic duct carcinoma of the gallbladder. CGP was then performed to determine the optimal chemotherapy regimen, which showed the following genetic findings: tumor nuclei percentage of 20%, a *KDM6A* nonsense mutation (E1376), a *KRAS* missense mutation (G12D), and a *MUTYH* splice site mutation (892–2A>G).

**Fig. 5:**
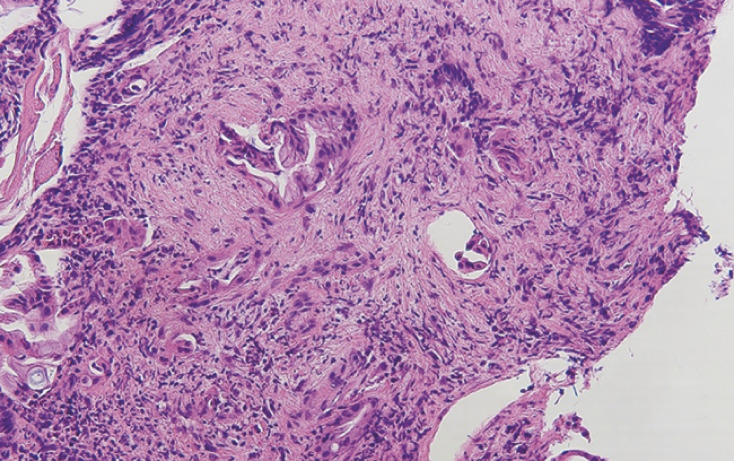
Histopathology revealed adenocarcinoma, with a tumor nuclei percentage of 20%.

Although POCS-guided biopsy enables target biopsy under direct visualization, there have been concerns regarding the relatively small sample volume that can be obtained. The SpyBite MAX forceps has been reported to improve tissue acquisition due to its significant size and shark tooth-like tip for better grasping 4 . Therefore, sufficient tissue sampling for CGP can be expected with the use of these forceps.

Endoscopy_UCTN_Code_TTT_1AR_2AD

## Notes

Endoscopy E-Videos https://eref.thieme.de/e-videos
*E-Videos* is an open access online section of the journal *Endoscopy* , reporting on interesting cases and new techniques in gastroenterological endoscopy. All papers include a high-quality video and are published with a Creative Commons CC-BY license. Endoscopy E-Videos qualify for HINARI discounts and waivers and eligibility is automatically checked during the submission process. We grant 100% waivers to articles whose corresponding authors are based in Group A countries and 50% waivers to those who are based in Group B countries as classified by Research4Life (see: https://www.research4life.org/access/eligibility/ ). This section has its own submission website at https://mc.manuscriptcentral.com/e-videos .
